# Editorial: Artificial intelligence and machine learning in pediatric surgery

**DOI:** 10.3389/fped.2024.1404600

**Published:** 2024-04-09

**Authors:** Rosa Verhoeven, Jan B. F. Hulscher

**Affiliations:** ^1^ Department of Surgery, Division of Pediatric Surgery, University Medical Center Groningen, University of Groningen, Groningen, Netherlands; ^2^ Department of Neonatology, Beatrix Children’s Hospital, University Medical Center Groningen, University of Groningen, Groningen, Netherlands

**Keywords:** artificial intelligence, machine learning, deep learning, pediatric surgery, neonatal and pediatric care, ELSA, surgical innovation

**Editorial on the Research Topic** Artificial intelligence and machine learning in pediatric surgery

## 1 Introduction

Cutting-edge technologies are leading to a profound transformation of healthcare (1, 2). Among the most promising advancements is the integration of artificial intelligence (AI) into the delicate and critical domain of pediatrics (3–5). We find ourselves at the threshold of a new frontier, envisioning how AI might revolutionize every step of the pediatric patient journey. In this Research Topic, we embark on an exploration of how AI may shape the future of pediatrics and pediatric surgery in particular.

## 2 Artificial intelligence and machine learning in pediatrics

The term “artificial intelligence” was coined by John McCarthy in 1955, defining it as “the science and engineering to make intelligent machines” (6). Over the years, AI has evolved into a vast field of computer science, levering technologies like machine learning to perform tasks that were once thought to require human intelligence, such as problem-solving, pattern recognition, and decision-making (7). In pediatrics, where healthcare providers often face intricate tasks demanding advanced human intelligence, AI has emerged as a transformative ally. The most recent technologies brought forward by AI can provide valuable support by analyzing extensive patient data and offering predictive insights which can be incorporated into early warning systems (8). Moreover, AI holds the potential to assist medical professionals in making precise diagnoses and suggesting personalized treatment recommendations (9–11). Beyond this, AI's capabilities extend into the operating theatre, where it can provide real-time information, robotic assistance, and procedural guidance, further advancing the field of pediatric surgery (12).

## 3 AI throughout the pediatric patient journey

This Research Topic delves into AI's potential future role in pediatrics, stretching even to the prenatal phase with Lin et al. exploring AI's potential in the detection of genomic mutations in congenital surgical diseases Lin et al. By navigating through a number of innovative deep learning models that identify and prioritize variations from big genomic data, they showed that AI can help to detect and understand the potential impact of mutations on disease development. This proactive approach enables timely interventions including preventive or corrective measures before or shortly after birth.

As we progress through the patient journey, AI's influence expands into the childbirth process, assisting healthcare professionals in monitoring and optimizing maternal and neonatal care. For instance, AI can play a pivotal role in analyzing progression data to predict the necessity of procedures like caesarean sections (13, 14). Post-birth, high-risk neonates undergo a series of diagnostic tests, including imaging scans and laboratory tests. Ongoing research into computer vision algorithms, utilizing convolutional neural networks for the analysis of medical images, holds the promise of more accurate diagnoses (15, 16). Future developments may even witness integration of sophisticated multi-modal algorithms, combining diverse data sources for highly precise predictions of specific medical conditions (17).

Where preventative measures fall short and critical diseases manifest, AI can step in to facilitate informed decision-making. An interesting example lies in the use of Behavioral Artificial Intelligence Technology, showcased in the study by Van Varsseveld et al. illustrating its potential in supporting physicians with end-of-life decision-making for preterm infants with surgical necrotizing enterocolitis.

Within the operating theatre, AI's potential in guiding surgical procedures becomes increasingly evident. While technologies like the use of indocyanine green fluorescence have proven successful in open-surgery Esposito et al., envisioning an AI-driven iteration might involve the integration of augmented reality (AR) technologies (18, 19). These would enable the visualization of crucial landmarks and surgical paths, providing invaluable guidance to surgeons. Another notable example highlighted in this Research Topic involves machine learning algorithms distinguishing between ventral and dorsal roots during selective dorsal rhizotomy using electro-neurophysiological characteristics Jiang et al. Furthermore, robotic-assisted surgery is not untouched by AI's transformative impact, offering surgeons greater precision, facilitating minimally invasive surgeries, and contributing to reduced incisions, pain, and faster recovery times for pediatric patients (20–22). Natural language processing might allow for automatic surgery reporting, streamlining documentation by extracting key information from the surgical procedure and generating detailed reports (23).

In postoperative care, AI can play a pivotal role by analyzing patient data to predict and prevent complications, optimizing recovery strategies, and personalizing rehabilitation plans. Moreover, it can streamline healthcare processes by optimizing appointment planning and enhancing overall efficiency in healthcare facilities. In essence, AI's integration into pediatrics encompasses a spectrum of technologies and applications, revolutionizing surgical practices and ultimately improving outcomes for pediatric patients and their families.

## 4 Ethical, legal and societal aspects (ELSA) of applying AI throughout the patient journey

Integration of AI in pediatrics and pediatric surgery brings along a great deal of interconnected ethical, legal, and social concerns, which are of particular relevance when caring for preterm and critically ill newborns and children.

As the article by Till et al. in this Research Topic explained, optimization of the developed algorithms can have a significant impact on its usability Till et al. By performing a thorough pre-processing procedure, they significantly improved the radiological detection of wrist fractures. Hence, before implementation, it is imperative to carry out iterative development and testing procedures to ensure optimal performance and clinical relevance of the model. While doing so, factors like algorithm bias and transparency must be meticulously considered to prevent disproportionate impacts on the vulnerable pediatric populations (24–26). Additionally, robust safeguards are essential to protect patient privacy. Taking a societal perspective, the deployment of AI-tools must transcend socio-economic boundaries, advocating for equal access to these transformative technologies (27). Simultaneously, a proactive approach to education is essential, empowering individuals with the knowledge necessary to use the tools optimally (28). The seamless integration of AI in pediatrics also necessitates the cultivation of trust, ensuring its acceptance as valuable aid rather than a potential source of apprehension (29, 30). On the legal front, AI prompts a reevaluation of medical liability and responsibility (31–33). Still, by navigating the social and legal dimensions conscientiously, a seamless integration of AI into pediatrics can be achieved, fostering advancements that benefit patients while upholding ethical, social, and legal standards.

## 5 Conclusion

In this Research Topic, we provide a glimpse of how AI could be possibly integrated into the pediatric patient journey. AI has the potential to be incorporated at any stage of this journey, catering to the specific needs and preferences of healthcare professionals, parents and patients alike. As we navigate the opportunities and challenges in this transformative era, the continuous evolution of AI holds the key to a future where technology becomes an even more indispensable ally in the pursuit of optimal pediatric care.
